# Freezing Behavior as a Response to Sexual Visual Stimuli as Demonstrated by Posturography

**DOI:** 10.1371/journal.pone.0127097

**Published:** 2015-05-20

**Authors:** Harold Mouras, Thierry Lelard, Said Ahmaidi, Olivier Godefroy, Pierre Krystkowiak

**Affiliations:** 1 EA 7273, Centre de Recherche en Psychologie: Cognition, Psychisme et Organisations, UFR de Sciences Humaines Sciences Sociales et Philosophie, Département de Psychologie, Université de Picardie Jules Verne, F-80000 Amiens, France; 2 EA 4559, Laboratoire de Neurosciences Fonctionnelles et Pathologies, UFR de Médecine, Université de Picardie Jules Verne, 3 rue des Louvels, F-80000 Amiens, France; 3 Service de Neurologie, CHU Amiens, Place Victor Pauchet, F-80054 Amiens Cedex 1, France; 4 EA 3300, Adaptations Physiologiques à l’Exercice et Réadaptation a l’Effort, UFR des Sciences du Sport, Université de Picardie Jules Verne, F-80025 Amiens, France; 5 Structure Fédérative de Recherche en Santé CAP-Santé, Université de Picardie Jules Verne, F-80000 Amiens, France, and Université de Reims-Champagne-Ardennes, F-51097 Reims, France; Leibniz Institute for Neurobiology, GERMANY

## Abstract

Posturographic changes in motivational conditions remain largely unexplored in the context of embodied cognition. Over the last decade, sexual motivation has been used as a good canonical working model to study motivated social interactions. The objective of this study was to explore posturographic variations in response to visual sexual videos as compared to neutral videos. Our results support demonstration of a freezing-type response in response to sexually explicit stimuli compared to other conditions, as demonstrated by significantly decreased standard deviations for (i) the center of pressure displacement along the mediolateral and anteroposterior axes and (ii) center of pressure’s displacement surface. These results support the complexity of the motor correlates of sexual motivation considered to be a canonical functional context to study the motor correlates of motivated social interactions.

## Introduction

Over the last two decades, the exponential development of modern neuroscience techniques has allowed investigation of neural processes and their peripheral correlates from new angles. Two important fields have recently emerged: (i) affective neurosciences [1], interested in identifying the neural processes involved in emotional and motivational processes and (ii) *social* neurosciences, focusing on the neural correlates of social information processing. These two scientific fields converge on several scientific issues, such as the identification of the neural and psychological mechanisms involved in the processing of emotional information expressed by congeners. Clearly, relationships between individuals firstly obey a motivational component of inter-attraction, which may be either positive or negative. This notion of inter-attraction can be considered in different functional contexts, such as *inter-individual* recognition, *attachment* or *affiliation* relationships. Therefore, in the fields of psychology and neuroscience, focus on socioaffective processes has shed light on *correspondences* within the *cognitive* and *neural* systems involved in the *production* of actions and their *perception* by another person. A canonical example of this correspondence is the discovery of mirror neurons within the motor cortex in primates, which increase their discharge frequency during both the *realization* of actions and the *observation* of the same actions performed by a peer [2]. Previous neuroimaging experiments on neural correlates of sexual motivation [3, 4] have shed light on the importance of the motivational component in the neurobehavioral model of sexual arousal [5, 6]. Fundamentally, emotion can be conceptualized as an action disposition, i.e. a tendency to do something [7, 8]. Emotion induces a context-dependent (e.g. approach *vs* avoidance) behavioral response mediated by automatic responses [9, 10]. Emotion modulates all steps of the motor response [11, 12, 13]. We therefore tend to approach what is acceptable to promote our well-being and our survival and avoid painful experiences in order to protect us from harm [14]. Lang et al. [15] classified emotional stimuli in terms of “approach-avoidance” behavior—indexed by the subject *vs* target distance—induced either as “appetitive” or “defensive”. According to these considerations, sexual motivation should incorporate sexual action tendencies and sexual action [16]. Several incentive motivation theories state that approach behavior is activated by appropriate incentives [17, 18, 19]. As noted by Both et al. [16], Bindra [18] defined, in his theory, *central motive state* as “a hypothetical set of neural processes that promote goal-directed actions in relation to particular classes of incentive stimuli”. In particular, an incentive is hypothesized to guide a response selection process via the excitatory (even priming) influence of the motive on locomotor actions. Therefore, according to Frijda [20], motivation will elicit a behavioral system (a sequence of potential actions) by appropriate external stimuli. Previous results have clearly supported this theoretical framework, as Both et al. [16] reported increased behavioral responsiveness and interest as well as spinal tendon reflexes in front of sexual videos as compared to neutral videos. Moreover, the magnitude of the spinal tendon reflexes increased with the level of arousal induced by sexual videos [21]. Stins et al. [22] reported more rapid initiation of gait towards a smiling as than towards an an angry face. Naugle et al. [23] reported no variation in walking speed towards highly unpleasant pictures compared to sexually explicit pictures for the first step, but a more rapid walking speed for the second step for sexual stimuli. Moving towards an unpleasant stimulus (“incongruent task”) creates an emotional conflict that interferes with the cognitive resources involved in the development of the movement [24], which seems to occur early (indexed by transient “freezing behavior”).

Although of major interest for the understanding of emotion/motor interactions, posturographic changes in motivational conditions remain largely unexplored. Posturography is a device used to determine displacement of the Center of Pressure (COP; [25]) in order to accurately quantify postural control. For this reason, posturography is increasingly used to evaluate small body sways while the participant remains stationary in front of an emotional scene. It is important to stress several important points to understand the contribution of posturography to our main research question. It is well known (and has been at the center of the exponential development of new scientific fields such as neuroeconomics) that emotion influences decision-making [26]. One possibility is that emotional biasing of action selection might indicate the effects of a Pavlovian system that innately regulates specified responses to reward—or punishment predictive stimuli [27]. As noted by Ly et al. [27], in humans, Pavlovian influences are evidenced by multiple studies [28, 29, 30, 31, 32] showing that reward potentiates behavioral activation, while punishment potentiates behavioral inhibition. The hypothesis that Pavlovian-like response tendencies account for emotional biasing of action selection by emotional faces had never been tested prior to the study by Ly et al. [27], which demonstrated that angry versus happy faces slowed instrumental approach versus avoidance responses. Critically, individual differences in this emotional biasing effect were predicted by individual differences in bodily freezing. Several recent studies have reported a general stiffening of the entire body when increasing the participant’s anxiety by introducing a postural threat [33, 34, 35, 36]. Hillman et al. [37] reported differences in body inclination in response to different emotional pictures, which were interpreted as an “approach-avoidance” type behavior supporting the model proposed by Lang et al. [15]. Stins and Beck [22] reported a decrease in the area of the COP path [22] and (ii) and several studies have demonstrated simultaneous freezing behavior and slowing of the heart rate [38, 39] in response to disgusting stimuli inducing avoidance. On the contrary, visual sexual stimuli are highly arousing and attractive but were recently demonstrated [37, 22] not to induce any differential COP path variation. In the fields of socioaffective psychology and neuroscience, sexual motivation now constitutes a widely recognized working model with very specific physiological variations, particularly adapted to explore the psychological and neural processes of motivated social interactions [40, 41]. As indicated above, in view of the close interactions between decision-making, motor processes and emotional processes, sexual motivation appears to be an ideal functional context for our primary objective, i.e. to quantify modulation of motor correlates in response to presentation of visual sexual stimuli in terms of approach-avoidance behavior concurrently with other physiological indices. Our main hypothesis was that an approach-type behavior would be observed in response to sexual stimuli compared to neutral visual stimuli.

## Material and methods

### Participants

Twenty-three healthy human males (mean age = 22.3 ± 3.7) with (i) no history of visual or motor impairment; (ii) no prior or current treatment for psychiatric or neurological disorders were included. All participants signed an informed consent form. The experimental procedures were in accordance with the ethical standards of the Helsinki declaration and were approved by the local ethics committee (Comité de Protection des Personnes Nord Ouest 2, Amiens, France).

### Stimulus materials

The study was based on a blocked design comprising three experimental conditions defined by three types of silent video clips validated in a previous neuroimaging study [3]: emotionally neutral documentary clips (N condition), sexually explicit films depicting heterosexual intercourse (S), and humorous clips, inducing a pleasant but nonsexual emotion (H condition). Three different video clips were presented for each of these three experimental conditions so that an experimental run comprised nine successive blocks. More precisely, the run comprised: (i) a 20-s white screen block; (ii) nine experimental blocks each lasting 50 s; (iii) a final 20-s white screen block. Each stimulation block was separated from the following by a 10-s white screen presentation to allow experimenters to discriminate effects of termination of the blocks from those of initiation of the blocks. The various experimental conditions were presented across subjects in random order. However, in order to avoid habituation effects, the same experimental condition was never assigned to two consecutive blocks.

### Posturographic assessments

Posturographic data were recorded using a Biopac MP150 system (Biopac Inc., Santa Barbara, CA). Movements of the center of pressure were recorded during the rest stance by a posturographic platform (Satel, Blagnac, France). Analog data from three strain gauges were recorded and movements of the center of pressure in the anteroposterior (AP) and mediolateral (ML) directions were computed by AcqKnowlege software (Biopac Inc., Santa Barbara, CA).

### Procedure

Firstly, participants stood barefoot in the middle of the force plate. They were asked to maintain a comfortable bipedal stance with their arms hanging relaxed alongside their body and their feet pointing 30° outwards. Visual stimuli were then presented 2 meters in front of the participants on a 17” screen. Participants were instructed to watch the images presented without any additional movement. Video clips were presented in random order.

### Data analysis

The mean postural response was calculated for each experimental condition. The following indices were calculated for each trial: (i) the mean COP position in the anteroposterior direction (COP-AP) and in the mediolateral direction (COP-ML), reflecting the extent to which a participant leaned towards the anterior or posterior direction or towards the left or the right during a 50-s trial; (ii) the area encompassed by displacements of the COP (COP-Area), corresponding to the surface area of the confidence ellipse containing 90% of the sampled COP positions; (iii) the amplitude of sway of the COP in the anteroposterior direction (Amp [COP]-AP) and in the mediolateral direction (Amp [COP]-ML), reflecting the maximal distance of COP displacement in the AP or ML directions; (iv) the standard displacement of the COP in the anteroposterior direction (SD [COP]-AP) and in the mediolateral direction (SD [COP]-ML), reflecting the variability of COP position in the AP or ML directions.

### Statistical analysis

These postural data were analyzed with a 3 (S, H, N experimental conditions video clips) repeated-measures analysis of variance (ANOVA) to study the effect of emotional stimuli on the postural response. When the F-ANOVA value was significant, the difference was tested using a pairwise Student’s t-test. The threshold for statistical significance was set to p <.05 for all analyses.

## Results

**Fig 1:**
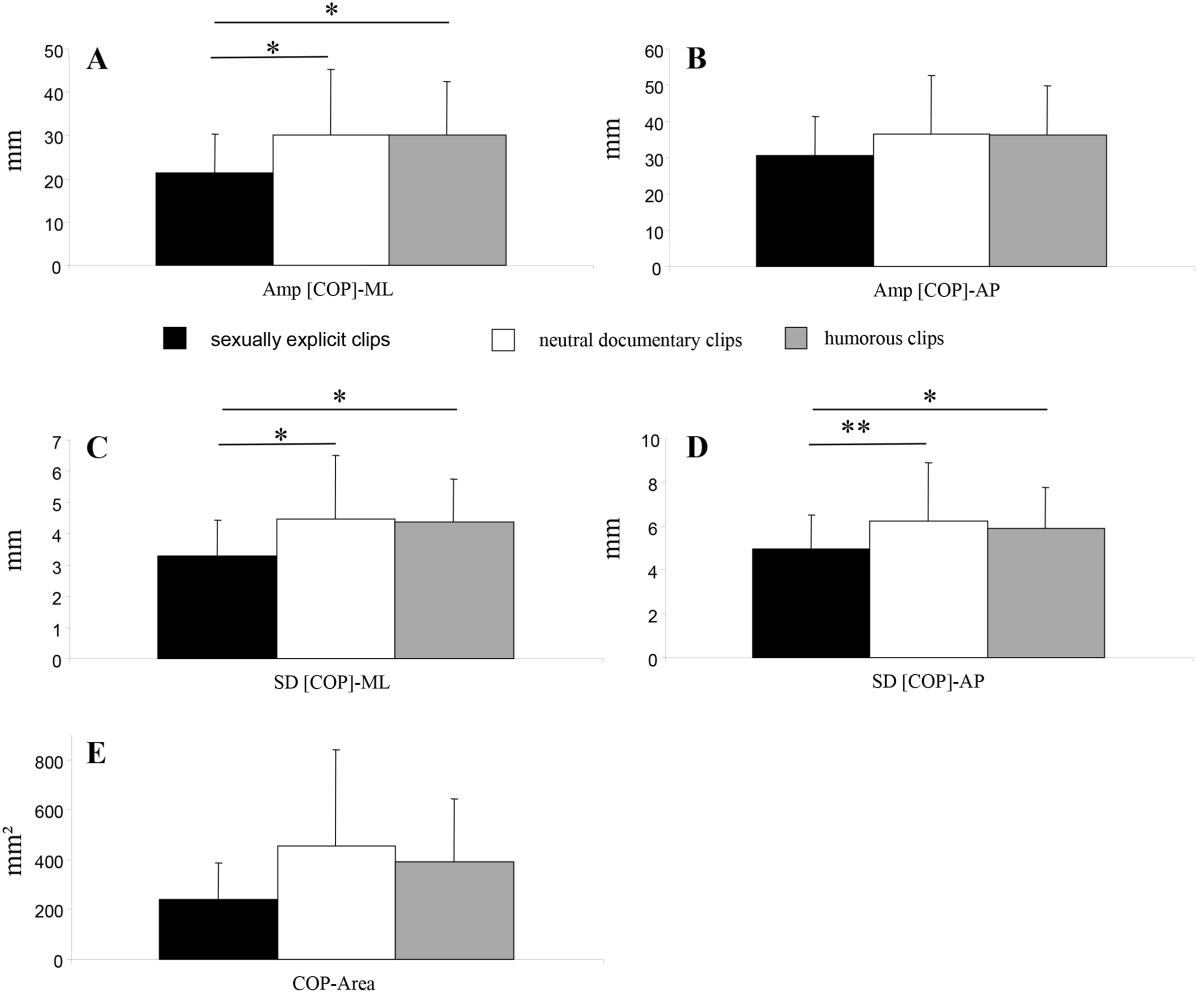
Mean ± SD for postural indices as a function of the stimulus (A) Amplitude of the sway of the COP in the mediolateral direction (Amp [COP]-ML) (B) Amplitude of the sway of the COP in the anteroposterior direction (Amp [COP]-AP) (C) Standard displacement of the COP in the mediolateral direction (SD [COP]-ML) (D) Standard displacement of the COP in the anteroposterior direction (SD [COP]-AP) (E) Area encompassed by displacements of the COP (COP-Area) Significant differences are indicated as follows: * p < 0.05, ** p<0.01 when comparing stimulus.

Postural responses to video clips are summarized in Table 1, Fig 1 and S1 Posturographic data in the three conditions.

**Table 1:** Mean motor indices in response to various stimuli.

	Appetitive	Neutral	Humorous
COP-ML^1^ (mm)	0.87 (1.96)	0.60 (1.62)	-1.45 (1.91)
Amp [COP]-ML^2^ (mm)	21.28 (9.08)	30.13 (15.14)	30.01 (12.36)
COP-AP^3^ (mm)	0.37 (3.59)	-0.79 (2.64)	1.13 (4.00)
Amp [COP]-AP^4^ (mm)	30.61 (10.58)	36.45 (16.25)	36.17 (13.63)
COP-Area^5^ (mm^2^)	241.09 (147.85)	456.13 (383.09)	391.23 (253.71)
SD [COP]-ML^6^ (mm)	3.30 (1.15)	4.47 (2.03)	4.36 (1.38)
SD [COP]-AP^7^ (mm)	4.95 (1.55)	6.22 (2.68)	5.91 (1.87)

^1^: COP position in the anteroposterior direction;

^2^: Amplitude of the sway of the COP in the mediolateral direction;

^3^: COP position in the mediolateral direction;

^4^: Amplitude of the sway of the COP in the anteroposterior direction;

^5^: Area encompassed by displacements of the COP;

^6^: Standard displacement of the COP in the mediolateral direction;

^7^: Standard displacement of the COP in the anteroposterior direction. Number in bracket is the Standard Deviation.

A one-way ANOVA demonstrated a significant effect of stimulus on COP-ML [F(1, 23) = 4.85, p = 0.01], due to a leftward shift during the presentation of H clips compared to S clips (p < 0.01) and to N clips p < 0.05). However, no significant differences were observed for COP-AP [F(1, 23) = 1.24, p = 0.29].

A significant effect of the stimulus was demonstrated on SD [COP]-ML [F(1, 23) = 3.81, p < 0.05], due to a lower value for SD [COP]-ML on presentation of S clips compared to N (p < 0.05) and H (p < 0.05) clips, while no significant difference was observed between H and N clips (p = 0.81). A significant effect of the stimulus was also demonstrated on SD [COP]-AP [F(1, 23) = 4.26, p < 0.05], due to a lower value for SD [COP]-AP on presentation of S clips compared to N (p < 0.01) and H (p < 0.05) clips, while no significant difference was observed between H and N (p = 0.49).

Although not significant, tendencies were observed for COP-Area [F(1, 23) = 3.07, p = 0.056] and Amp [COP]-AP [F(1, 23) = 2.62, p < 0.08]. A significant effect of the stimulus was demonstrated on Amp [COP]-ML [F(1, 23) = 3.69, p < 0.05], due to a lower value for Amp [COP]-ML on presentation of S clips compared to N (p < 0.05) and H (p < 0.05) clips, while no significant difference was observed between H and N (p = 0.97).

## Discussion

The primary objective of this study was to quantify modulation of motor correlates in response to presentation of visual sexual stimuli in terms of an approach-avoidance behavior with the main hypothesis of an approach-type behavior in response to sexual visual stimuli compared to neutral visual stimuli.

The main result of this study is the demonstration of a differential modulation of motor processes according to the emotional and motivational nature of the stimuli in contrast with other physiological responses, such as cardiac or electrodermal responses. Therefore, the perceived motor responses could be central in motivated social relationships for the observer. In this study, we investigated postural changes induced by sexually explicit videos in order to study motivated social interactions. Our main hypothesis was that an approach-type behavior would be observed in response to sexual stimuli compared to neutral visual stimuli. However, the results of this study do not support this hypothesis, but support a a freezing-type response in response to sexually explicit stimuli compared to other conditions, as demonstrated by significantly decreased SDs (along both the ML and AP axes) and COP displacement area, indicating a lower amplitude of movement of the participants in response to visual sexual stimuli. Previous studies in the literature have reported a modulation of postural control in various emotional situations. Most of these studies have reported that humans adopt a freezing strategy in response to aversive visual images [39, 22, 37]. Interestingly, a recent study [42] used video clips for the first time film to study postural changes in response to pleasant, unpleasant and neutral visual stimuli. The authors reported a defense response (as reflected by reduced body sway and heart rate deceleration) in response to unpleasant compared to both pleasant and neutral videos. A recent study reported modulation of a freezing strategy by the arousal dimension of the stimuli and not by their valence [43]. Sexual stimuli are the most arousing stimuli of the category of positive emotional and motivational stimuli. Our results can be interpreted in the light of a recent review of the literature focusing on the concept of freezing as freezing is usually considered to be thisa threat-related defense strategy [44]. At first sight, our behavioral responses appear to be incongruous in response to motivating stimuli. Haagenars et al. [44] reported immobility as the main characteristic of other types of response, such as orienting or tonic immobility, behavioral inhibition and reported that immobility may be difficult to differentiate from freezing. According to these authors, freezing may have been inconsistently reported as orienting, avoidance, vigilance, attentive immobility and anxiety. The results of the present study would therefore be consistent with the development of a freezing-like strategy following an increase in the arousal dimension of the stimuli. However, this can also be linked to a change in the attention paid to the specific instructions for posturographic recording that requires the subject to remain motionless for 12 seconds while facing the stimulus.

Moreover, Hagenaars et al. [42] reported an early (1–2 s after stimulus onset) freezing behavior in response to unpleasant films, which highlights the importance of including time-courses in defense reaction studies. This early freezing component of the response associated with a body position is optimal for concealment from a predator and optimally prepares for action [45]. Consequently, this type of response is considered to be part of a fear-type response. Therefore, our results can shed light on a certain paradox of sexual motivation which can certainly be associated with hedonic and positively valanced emotional aspects, but also with the appearance of a certain anxiety which is more negatively valanced. For example, Roelofs et al. [46] found that freezing in response to angry faces was related to an anxiety state. However, on a broader review of the literature, motor, neural and psychological processes appear to be more complex, as body sway reductions were observed in response to both unpleasant and pleasant pictures [39]. The authors of this very important study rightly suggested that “their baby and family pictures may have elicited a predisposition to social bonding and that the pre-activation of muscles involved in the anterior-posterior displacement could reflect preparation for processes like attachment and reduction of social distance. This view is consistent with animal models that posit that the organism prepares for action during freezing [47, 48]. For example, more recent human models suggest that freezing is a brief orienting response preceding action [49]. Our results are also in accordance with other studies supporting the idea of motor preparation processes in response to visual sexual stimuli. Several studies reported in various tasks increased reaction time in response to visual sexual stimuli compared to neutral ones which supports the idea of the recruitment of supplementary cognitive resources(including motor preparation processes) by motivational stimuli [50, 51, 52, 53]. Moreover, our result are in accordance with a much more recent study [54] which suggests that the recruitment of neural motor networks is an integral part of the sexual response. Using transcranial magnetic stimulation, the authors demonstrate that visual sexual stimuli corresponding with one’s sexual orientation increase the excitability of the motor cortex.

Basically, the hypothesis of a certain behavioral inhibition is also compelling with our data. One possibility to disambiguate this hypothesis from the one of motor preparation would be to use other appetitive states such hunger to see if postural responses are modulated by hunger.

In conclusion, our results support the value of sexual motivation as an excellent functional contact to study and identify the motor, neural and psychological processes involved in social interactions. These results support the complexity of the processes involved, particularly regarding the body language involved in our motivated social interactions. We believe that further studies are required particularly to investigate the core time-course of the motor correlates of sexual motivation.

## Supporting Information

S1 Posturographic data in the three conditions Main posturographic data recorded in the appetitive, neutral and humorous condition. (XLS)
